# Gene Expression Signature Associated with Clinical Outcome in ALK-Positive Anaplastic Large Cell Lymphoma

**DOI:** 10.3390/cancers13215523

**Published:** 2021-11-03

**Authors:** Camille Daugrois, Chloé Bessiere, Sébastien Dejean, Véronique Anton-Leberre, Thérèse Commes, Stephane Pyronnet, Pierre Brousset, Estelle Espinos, Laurence Brugiere, Fabienne Meggetto, Laurence Lamant

**Affiliations:** 1 Inserm, UMR1037 CRCT, F-31000 Toulouse, France; daugrois.camille@inserm.fr (C.D.); stephane.pyronnet@inserm.fr (S.P.); brousset.p@chu-toulouse.fr (P.B.); estelle.espinos@inserm.fr (E.E.); 2 Université Toulouse III-Paul Sabatier, UMR1037 CRCT, UMR5071 CNRS, F-31000 Toulouse, France; 3 Laboratoire d’Excellence Toulouse Cancer-TOUCAN, F-31037 Toulouse, France; 4 Institut de Médecine Régénératrice et de Biothérapie, Inserm U1183, CHU Montpellier, F-34295 Montpellier, France; chloe.bessiere@inserm.fr (C.B.); therese.commes@inserm.fr (T.C.); 5 Institut de Mathématiques de Toulouse, UMR 5219 CNRS, Université Toulouse III-Paul Sabatier, F-31062 Toulouse, France; sebastien.dejean@math.univ-toulouse.fr; 6 Laboratoire d’Ingénierie des Systèmes Biologiques et des Procédés (LISBP), UMR INSA/CNRS 5504/INRA 792, INSA Batiment Bio 5, 135, Avenue de Rangueil, CEDEX 4, F-31077 Toulouse, France; veronique.anton@institutfrancais-chine.com; 7 Department of Pediatric Oncology, Institut Gustave-Roussy, F-94805 Villejuif, France; laurence.brugiere@gustaveroussy.fr

**Keywords:** ALK^+^ ALCL, predictive signature, relapse, clinical outcome

## Abstract

**Simple Summary:**

Anaplastic large cell lymphomas associated with ALK translocation have a good outcome after CHOP treatment; however, the 2-year relapse rate remains at 30%. Microarray gene-expression profiling, high throughput RT-qPCR, and RNA sequencing of 48 ALK-positive anaplastic large cell lymphoma (ALK^+^ ALCL) samples obtained at diagnosis enable the identification of genes associated with clinical outcome. More particularly, our molecular signatures indicate that the FN1 gene, a matrix key regulator, might also be involved in the prognosis and the therapeutic response in anaplastic lymphomas.

**Abstract:**

Anaplastic large cell lymphomas associated with ALK translocation have a good outcome after CHOP treatment; however, the 2-year relapse rate remains at 30%. Microarray gene-expression profiling of 48 samples obtained at diagnosis was used to identify 47 genes that were differentially expressed between patients with early relapse/progression and no relapse. In the relapsing group, the most significant overrepresented genes were related to the regulation of the immune response and T-cell activation while those in the non-relapsing group were involved in the extracellular matrix. Fluidigm technology gave concordant results for 29 genes, of which FN1, FAM179A, and SLC40A1 had the strongest predictive power after logistic regression and two classification algorithms. In parallel with 39 samples, we used a Kallisto/Sleuth pipeline to analyze RNA sequencing data and identified 20 genes common to the 28 genes validated by Fluidigm technology—notably, the *FAM179A* and *FN1* genes. Interestingly, FN1 also belongs to the gene signature predicting longer survival in diffuse large B-cell lymphomas treated with CHOP. Thus, our molecular signatures indicate that the FN1 gene, a matrix key regulator, might also be involved in the prognosis and the therapeutic response in anaplastic lymphomas.

## 1. Introduction

Anaplastic large cell lymphoma (ALCL) is a rare type of T-cell lymphomas, accounting for approximately 3% of adult non-Hodgkin lymphomas and 10 to 20% of childhood lymphomas [1]. Systemic ALK-positive ALCLs (ALK^+^ ALCL), associated with the translocation of the *Anaplastic Lymphoma Kinase* (*ALK*) oncogene, are considered a distinct entity in the WHO classification [1,2]. Chemotherapy treatments are based on cyclophosphamide, vinca-alkaloids, doxorubicin, and corticosteroids in both adults and children, and high-dose methotrexate in children. ALK^+^ ALCL tumors have a better outcome than other aggressive non-Hodgkin lymphomas, with a 5-year overall survival (OS) rate of 70% for adults and >90% for children [3,4,5,6,7,8,9,10]; however, the 2-year relapse rate remains at 30% [3,4,5,6,7,8,10,11]. To develop patient-tailored therapy strategies, we first need to be able to stratify patients according to risk factors. Several prognostic factors have been recently described for paediatric ALK^+^ ALCLs, including the detection of minimal disseminated disease (MDD) [12], in bone marrow or blood combined with antibody titers against ALK [13,14,15,16]. The histological subtype variant (versus the common morphology) is also associated with the prognosis in ALK^+^ ALCLs, at least in children [17]. However, the stratification of patients according to these prognostic factors has yet to be validated in randomized trials. We profiled gene expression in pre-treatment biopsies from non-relapsing and relapsing patients with ALK^+^ ALCL to provide an additional indicator that could help to identify patients with a high risk of relapse and those of low risk who could benefit from a therapy reduction. Several techniques were used to identify differentially expressed genes, i.e., micro-arrays and RNA-sequencing. Then, Fluidgim technology and the Kallisto/Sleuth pipeline helped us to cross-validate candidate genes.

## 2. Materials and Methods

### 2.1. Patient Characteristics and Tumor Samples

The diagnosis of ALK^+^ ALCL was based on morphologic and phenotypic criteria, as described in the 2001 and 2008 WHO classifications [1,2]. Histopathological and immunostaining results were reviewed by a national (the French Lymphopath Network) or international panel of pathologists [17]. Only cases with at least 50% lymph node involvement, assessed by CD30 staining frozen biopsies, and good RNA integrity (≥7) were selected from our tumor bank. The cohort consisted of 48 systemic ALK^+^ ALCL tumor samples obtained at the time of diagnosis between 1994 and 2009 (Table 1 and Table S1). The median follow-up was 58 months (4.8 years). Eighteen additional cases of systemic ALK^+^ ALCL with available frozen material at the time of the diagnosis were retrieved from our tumor bank and used as an independent validation cohort. The patients were all treated with intensive chemotherapy, most of them according to the ALCL99 protocol and stratified on clinical factors [18]. Others were treated according to malignant histiocytosis protocols (HM89 and HM91) [19] or with ACVBP (doxorubicin, cyclophosphamide, vindesine, bleomycin, and prednisone). Patient samples were obtained after informed consent in accordance with the Declaration of Helsinki, and approval was received from the relevant ethics committees. All samples were stored at the «CRB Cancer des Hôpitaux de Toulouse» collection. In accordance with French law, the CRB cancer collection has been declared to the Ministry of Higher Education and Research (DC 2009-989) and a transfer agreement has been obtained (AC-2008-820) after approbation by ethical committees. Clinical and biological annotations of the samples have been declared to the CNIL (Comité National Informatique et Libertés).

### 2.2. Microarrays

Two µg of total RNA from 48 samples were used for hybridization to HG-U133Plus 2.0 GeneChips (54,675 probe sets; Affymetrix, Santa Clara, CA, USA), as previously reported [20]. For each outcome group, gene expression data were extracted and normalized using the GCRMA method [21,22] with the *gcrma* package for Bioconductor 3.14 (http://bioconductor.org, accessed on 26 September 2021). Then, the data were filtered (using the *genefilter* package) to eliminate probe sets whose expression values were too low and that could therefore be difficult to reproduce using very sensitive methods such as quantitative RT-PCR (RT-qPCR) [23]. Thus, only probes with normalized log2-transformed expression levels higher or equal to 5 within at least one outcome group were considered. Finally, a differential analysis was carried out using the Empirical Bayes method with the *limma* package [24], and the list of genes significantly discriminating between relapsing and non-relapsing groups was retained with a False Discovery Rate (FDR) [25] adjusted *p*-value of <0.05 and a fold change (FC) of at least ±2. Overrepresented biological functions and pathways (biological processes, cellular components and molecular functions) that were associated with the differentially expressed genes were assessed using the *GOstats* [26] package in Bioconductor. 

### 2.3. RNA-Sequencing Data

From the 48 patient biopsies, 39 (18 relapsing and 21 non-relapsing) were retained for RNA-sequencing analysis. After ribodepletion (NEBNext^®^ rRNA Depletion HMR kit from NEB), RNA-seq libraries were prepared using NEBNext^®^ Ultra™ II Directional RNA Library Prep Kit for Illumina^®^ (NEB) and sequenced with Novaseq 6000 (ILLUMINA). The libraries’ preparations were realized following the manufacturer’s recommendations then sequenced to obtain 2 × 200 million 150-base reads per sample.

### 2.4. Validation of Microarray Signature Using High-Throughput Quantitative PCR Method

The oligonucleotide primer pairs used for the qPCR were designed with PrimerBLAST (http://www.ncbi.nlm.nih.gov/tools/primer-blast/, accessed on 26 September 2021) to target the CDS region of the variants detected by the selected Affymetrix probe sets. Primer Tms were calculated using Schildkraut and Lifson’s 1965 salt-correction formula and Breslauer’s 1986 table of thermodynamic parameters. The primer design was performed to avoid genomic DNA (gDNA) amplification. gDNA amplification was controlled during the primer validation and in the high-throughput qPCR by adding a positive control of gDNA (G147, Promega^®^, Charbonnières-les-Bains, France) and by a valid prime assay, which accurately corrects all reactions in BioMark Array for signals derived from gDNA [27]. Primer sequences are reported in the Table S2. 

PCR specificity was verified by assessing the melting curves of each amplification product. Primer efficiency has been tested on a pool of samples by standard qPCR (Table S2) prior to high-throughput qPCR. All qPCR assays were performed in duplicate. After a pre-amplification of cDNA, validation of the differentially expressed genes was performed using 96.96 Dynamic Arrays for the BioMark™ system (Fluidigm CorporatioSan Francisco, CA, USA) [23] according to manufacturer’s instructions. An initial data analysis was performed with the Fluidigm real-time PCR analysis software using the linear derivative baseline correction, a quality correction set to 0.65, and the User (Detectors) Cycle Threshold. The cq (quantification cycle) ranged from 6.7 to 22.7 which signed for a successful experiment [28]. The cts for undetectable targets were set at 31. The mean expression of MLN51 and TBP, selected as the best housekeeping genes using Genorm^®^ and Normfinder^®^ with the R package *NormqPCR*, was used as a normalization factor to calculate ∆Cq values (1):

[∆Cq_gene of interest_ = mean duplicate Cq_gene of interest_ − mean duplicate (Cq_MLN51_, CqTBP)] (1)

The −ΔCq values were used for heatmap and boxplot (*Beeswarm* package, https://rdrr.io/cran/beeswarm/man/beeswarm.html, accessed on 26 September 2021) generation by using the R software (version 3.1.2). The validation of the microarray signature was conducted using ΔCq values after an assessment for, first, an adjusted *p*-value from the Wilcoxon test, followed by a Benjamini–Hoechberg correction lower than 0.05, then a Pearson’s correlation between high-throughput qPCR and microarray data greater than 0.7.

### 2.5. Clinical Outcome Based on High-Throughput RT-qPCR Data

The validation of microarray signatures was carried out using ΔCq values after assessments for *p*-values from a Wilcoxon test followed by a Benjamini–Hoechberg correction. The selection criteria were a *p*-value lower than 0.05 and a Pearson’s correlation between high-throughput RT-qPCR and microarray data greater than 0.7. A two-step scheme to select the genes best discriminating between outcomes was established using ΔCq values. The first step involved two complementary methods based on distinct approaches that reach the same goal [29]: Random Forest (RF, using the *random Forest* package [30], *n* = 500 trees) and Partial Least Squares Discriminant Analysis (PLS-DA, using the *DiscriMiner* package, http://cran.r-project.org/web/packages/DiscriMiner/index.html, accessed on 26 September 2021). For RF, 70% of the cohort (34 cases) formed a training set and the remaining 14 tumors formed the test set. Each set had approximately the same proportion of relapsing and non-relapsing cases as the whole cohort. A PLS-DA algorithm was associated with leave-on-out cross-validation. We selected the top five genes from each method, ranked by significance (using the Gini index and VIP [variable importance for the projection] index, respectively). These index values represent a quantitative statistical parameter ranking genes according to their ability to discriminate between the two outcome groups. Selected genes were then used to develop a logistic regression model with a backward selection method using relapse as the outcome variable. 

### 2.6. Transcripts Quantification and Differential Expression Analysis

The Kallisto v0.44.0 pseudo-alignment method [31] was used to quantify transcript abundances directly from the raw RNA-seq FASTQ files. This method, based on the pseudo alignment for rapid and accurate quantification, was performed with a 100 bootstrap value, using a transcriptome index constructed from the Ensembl project’s transcriptome v91. Spring Cloud Sleuth version 0.30.0 [32] was then used within R for differential expression analysis at the gene level (gene mode = TRUE) with an aggregation of the transcript abundances by Ensembl’s gene ID (aggregation_column = ‘ens_gene’). Poorly covered genes (read count <10 in more than half of the samples) were removed before any further analysis. Genes were then defined as differentially expressed (DE) depending on the corrected *p*-value (qval, adjusted *p*-values using the Benchamini–Hochberg method) from the Sleuth statistical test. We tested both the Wald test (WT) and the likelihood ratio test (LRT), which is more stringent. 

## 3. Results

### 3.1. Clinical and Pathological Characteristics of Patients

Among the 48 patients (Table 1 and Table S1 and ref [33]), 31 were male and 17 were female. Most patients were children or young adults less than 22 years (*n* = 39). The median age at diagnosis was 12.5 years (range: 2–50 years). According to the Ann Arbor classification, 30 patients had advanced stage III or IV disease, and 18 had localised stage I or II disease. Twenty-two tumors were classified as common type and 26 as morphologic variants. The *ALK* gene was fused to the *NPM* gene in 44 tumors and to the *TPM3* gene in the other cases, which corresponded to the different ALK staining patterns [17]. After front-line multi-agent chemotherapy, 45 patients achieved complete remission. Three patients progressed during treatment (median: 7.2 months; range: 2.4–16.5 months), and 23 patients relapsed within 16.5 months of diagnosis: these were all assigned to the relapsing group. Twenty-two remained disease-free after a period of at least three years and were included in the non-relapsing group.

### 3.2. Molecular Signatures from Microarray Data Associated with Clinical Outcome 

**Figure 1:**
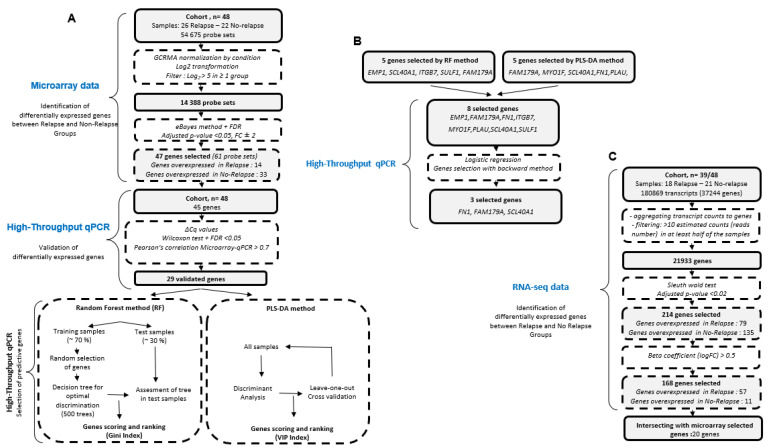
Workflow of development and validation. (**A**): Microaaray data and high- throughput qPCR workflow. (**B**): Gene selection using high- throughput qPCR. (**C**): Identification of differentially expressed gene between relapse and no-relapse groups using RNA sequencing.

**Figure 2:**
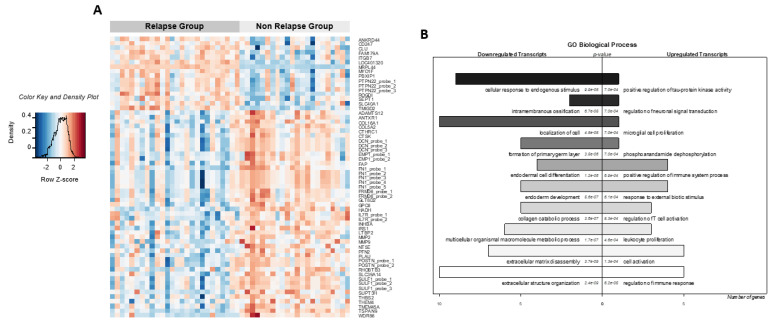
Molecular signature associated with clinical outcome and Gene Ontology Biological Process enrichment. (**A**) Heatmap of microarray data showing the 47 deregulated genes in “relapsing” (*n* = 26, dark grey) compared to “non-relapsing” samples (*n* = 22, light grey). Each column represents a sample, and each row a probe set or gene. The expression level of each probe set was standardized by subtracting that probe set’s mean expression from its expression value and then dividing this by the standard deviation across all the samples. This scaled expression value, designated as the row Z-score, was plotted using a red–blue color scale with red indicating high expression and blue indicating low expression. (**B**) Enrichment of these deregulated genes within the Gene Ontology (GO) categories with the 10 most-listed GO biological processes categories (*p* < 0.01). The number of probe sets downregulated or upregulated in “relapsing” specimens is represented below. The *p*-values of each GO category are reported on the graph.

Based on microarray data, a supervised method was used to find the most significant differentially expressed genes between relapsing and non-relapsing tumors. Using a significance level of corrected *p*-value <0.05 and a cut-off fold change of ±2 (Figure 1A), we generated a list of 47 significantly discriminating genes (61 probes), using the 14,388 probe sets that had a log2-transformed expression level ≥5 within at least one group (Figure 2A, Table 2, orange columns). Among the 47 genes, 14 genes were overexpressed in the relapsing group while 33 genes were overexpressed in the non-relapsing group (Figure 2A, Table 2, orange columns). 

The most significantly overrepresented GO terms (biological processes, Figure 2B and Table S3) in the relapsing group were related to the regulation of the immune response: *clusterin* (CLU logFC:1.07; adjusted *p* value: 3.87 × 10^−2^), integrin beta7 *ITGB7* (logFC: 1.47; adjusted *p* value: 4.14 × 10^−2^), the tyrosine phosphatase *PTPN22* (logFC: 1.34; adjusted *p* value: 2.29 × 10^−2^), the unconventional myosin *MYOF1 genes* (logFC: 1.04; adjusted *p* value: 2.75 × 10^−3^) and T−cell activation (the CD3 zeta chain gene called as *CD247*; logFC: 1.14; adjusted *p* value: 4.53 × 10^−2^), and *TMIGD2* (logFC: 1.97; adjusted *p* value: 1.28 × 10^−7^), a new member of the T−cell costimulatory/coinhibitory B7/CD28 families. In the non−relapsing group, highly expressed genes were involved in extracellular matrix (ECM) organization and disassembly: *FN1* (fibronectin1; logFC: 1.25; adjusted *p* value: 1.18 × 10^−2^), *DCN* (decorin, logFC: 1.12; adjusted *p* value: 3.59 × 10^−2^), *FAP* (fibroblast activating protein, logFC: 1.59; adjusted *p* value: 1.68 × 10^−2^), *ADAMTS12* (logFC: 1.39; adjusted *p* value: 2.21 × 10^−2^), *MMP2* (logFC: 1.57; adjusted *p* value: 4.57 × 10^−2^), *MMP9* (logFC: 1.55; adjusted *p* value: 4.72 × 10^−2^), and different collagen family members such as the *COL16A1*, *COL5A2*, *ANTRX1*, *CTSK*, and *CTHRC1* genes.

To validate the signatures of 47 genes, RT-qPCR using high-throughput Fluidigm^®^ technology was performed on all biopsies. Expression of the *CD247* and *PFN2* genes was not taken into account in the final analysis because these primers formed dimers. Among the 45 remaining genes, 29 gave concordant results with an adjusted *p*-value of <0.05 and a Pearson’s correlation coefficient of >0.7 (Figure 1A, Table 2: blue columns; *ANTXR1, CTHRC1, CTSK, DCN, EMP1, FAM179A, FAP, FN1, FRMD6, GLT8D2, GPC6, IL7R, INHBA, IRS1, ITGB7, MMP2, MMP9, MYO1F, NT5E, PLAU, POSTN, PTPN22, ROGDI, SLC39A14, SLC40A1, SULF1, THBS2, TMEM45A, TSPAN9*).

### 3.3. Identification of a Minimum Set of Genes Associated with Clinical Outcome 

**Figure 3:**
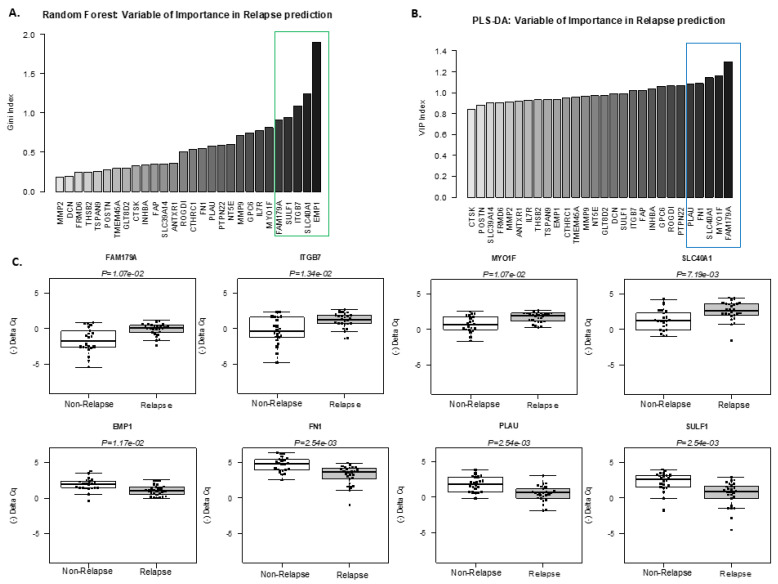
Selection of best predictive genes for outcome stratification by Random Forest and PLS-DA analysis of high-throughput RT-qPCR data. Relative importance of genes that discriminated between “relapsing” and “non-relapsing” groups in high-throughput RT-qPCR data. The bar plots show the mean Gini index of each gene from Random Forest classification (**A**) and variable importance in the projection (VIP) of the PLS-DA method with (**B**) larger values (to the right of the graph) indicating a more important gene within the model. The five top genes are highlighted by a grey box. (**C**) Box plots and strip-charts showing high-throughput qPCR quantification of the 8 selected genes in “relapsing” (grey, *n* = 26) and “non-relapsing” (white, *n* = 22) samples. Statistical significance was calculated using the Wilcoxon test followed by a Benjamini and Hoechberg correction. Expressions are given as (−∆Cq).

Random Forest (RF) and Partial Least Squares Discriminant Analysis (PLS-DA) are two powerful tools for analysing microarray data. Because these two algorithms can highlight essential variables in a dataset, we used them as classification algorithms on high-throughput RT-qPCR data to identify the minimum set of genes whose expression in primary tumors is associated with clinical outcome (Figure 1A). Using RF analysis, the optimal gene classifier consisted of five genes: *EMP1*, *SCL40A1*, *ITGB7*, *SULF1,* and *FAM179A*, ranked according to their variable importance in the model (Figure 1B and Figure 3A). PLS-DA algorithms also gave an optimal gene classifier consisting of five genes in rank-order: *FAM179A*, *MYOF1*, *SCL40A1*, *FN1,* and *PLAU* (Figure 1B and Figure 3B). Therefore, RF and PLS-DA selected a total of 8 genes that could help classify relapsing and non-relapsing patients (Figure 1B and Figure 3C). We then tried to reduce the number of genes even more. Using a logistic regression on the ΔCq expression from using high-throughput Fluidigm^®^ technology with these 8 genes, we identified a set of 3 genes (Figure 1B, Table S4): *FN1*/fibronectin 1, *FAM179A* (family with sequence similarity 179, member A), and *SCL40A1*/ferroportin-1. For these three genes, data generated by microarray, Fluidigm^®^, and standard RT-qPCR showed an excellent consistency (R^2^ > 0.89, Figure S1). Overexpressions of the 3 genes are validated in relapse groups using an independent cohort (*n* = 18, Figure S2). Finally, since all are located on chromosome 2 (2q34, 2p23.2 and 2q32, respectively), we verified that their differential expression was not related to the gain or deletion of their loci by high-resolution CGH array.

### 3.4. Transcripts Quantification with Pseudo-Alignment and Differential Expression Analysis by Total RNA-Sequencing 

To find a gene’s signature from another transcriptomic technique, 18 relapsing and 21 non-relapsing tumors over the 48 patient biopsies (39/48 samples) were sequenced. Using full RNA 150-bp paired-end sequencing data (median of 507 million reads per patient), gene expression was quantified with Kallisto, a fast pseudoalignment-based method used to obtain transcript quantification from RNA sequencing data [31]. Genes differentially expressed (DE) between relapsing and non-relapsing conditions were selected with Sleuth, which is a program for the differential expression analysis of RNA-Seq experiments for which transcript abundances have been quantified with Kallisto [32]. With a corrected *p*-value < 0.02, 214 genes were found as DE between the two groups (relapse and no relapse) with the statistical Wald Test (WT, Figure 1C) and 62 with the more stringent Likelihood Ratio Test (LRT) (Table S5), which is a statistical test of the goodness-of-fit between two models. We finally retained the Wald Test’s most extensive list for further analysis because it gives a ‘beta’ value (size effect) that can be compared to logFC. Thus, finally, 168 genes having an absolute log2 FC between relapse and no-relapse groups greater than 0.5 and a *p*-value lower than 0.02 [34] were selected (Figure S3). After intersecting these 168 DE genes with the 47 significantly discriminating genes previously found with the microarray technique (*p* value < 0.05), 20 common genes were highlighted (*ANTXR1, CTHRC1, DCN, FAM179A/TOGARAM2, FAP, FN1, FRMD6, GLT8D2, INHBA, IRS1, ITGB7, MYO1F, NT5E, PLAU, PBXIP1, PTPN22, SLC39A14, SULF1, THBS2,* and *LTBP2*) (beta value/WT “log2FC” estimator greater than 0.5 and *p* value < 0.02; Table 2: green columns, red lines) including 5 and 15 genes overexpressed in relapse and no-relapse groups, respectively (Figures S4 and S5). On these 20 genes, 18 (*ANTXR1, CTHRC1, DCN, FAM179A/TOGARAM2, FAP, FN1, FRMD6, GLT8D2, INHBA, IRS1, ITGB7, MYO1F, NT5E, PLAU, PTPN22, SLC39A14, SULF1,* and *THBS2*) have also been validated with high-throughput Fluidigm^®^ technology (Figure S5). Among them, the *FAM179A* and *FN1* genes were already selected after logistic regression on the ΔCq expression from the high-throughput Fluidigm^®^ technology data (Table S4). 

## 4. Discussion

Although systemic ALK^+^ ALCL are highly chemosensitive tumors, with a 5-year OS rate of 80%, 30% usually experience relapse within the year following the end of treatment. Moreover, these “early” relapses are associated with a bad prognosis [35]. In the present study, we sought to identify a molecular signature that was associated with clinical outcome (relapse/progression versus non-relapse) in systemic ALK^+^ ALCL. From a cohort of 48 tumor samples obtained at diagnosis, our supervised analysis based on micro-array data identified 47 genes that significantly discriminated the two groups. Twenty of them were also found to be differentially expressed by RNA sequencing, supporting their biological significance. 

In the microarray molecular signature of the relapsing group, the most significant *p*-values included the overexpression of six genes (*FAM179A, ITGB7, MYOF1, SLC40A1* or *Ferroportin-1, PTPN22,* and *ROGDI*). Many of the genes that were overrepresented and up-regulated in this group were implicated in the regulation of the immune response and in T-cell activation and proliferation. For the non-relapsing group, *INHBA, GPC6, SULF1, FN1*, *PLAU,* and *FAP* were the top six genes overexpressed with the most significant *p*-values. Eight of them were also differentially expressed using RNA-seq analysis (*FAM179A, ITGB7, MYOF1,* and *PTPN22* in the relapse group, and *FAP, FN1,* and *INHBA, SULF1* in the no-relapse group). Within the genes overexpressed in this non-relapsing microarray signature, there was a statistically significant overrepresentation of genes involved in extracellular matrix (ECM) deposition and organization. The ECM is a highly dynamic structure which is constantly being remodeled and, in the appropriate context, might restrain malignant tumor progression. Although excessive ECM deposition could hinder the diffusion of therapeutic agents [36] and play a role in cell adhesion-mediated drug resistance [37], proteases secreted by tumor cells and/or cells of the micro-environment could lead to its structure breakdown and influence the tumor cell response to chemotherapy. Furthermore, although proteases have long been considered as cancer-promoting factors, recent studies have revealed that they can also elicit tumor-suppressive effects through the stimulation of apoptosis or the inhibition of angiogenesis [38]. This ECM signature probably reflects a strong ECM deposition that could be associated with a peculiar tumor microenvironment less favorable for tumor cells. Interestingly, 19 of the 33 overexpressed genes in the microarray non-relapsing signature and 13 out of the 16 genes in the RNA sequencing non-relapsing signature also belong to the “stromal-1 signature” (including *FN1*) associated with a better EFS and OS in diffuse large B-cell lymphomas (DLBCL) treated by CHOP or R-CHOP [39]. Thus, our molecular signatures point out that the ECM could be involved in the prognosis and the therapeutic response in ALCL, as it has already been suggested in DLBCL. 

## 5. Conclusions

We have identified a minimum set of genes whose expression could help to predict clinical outcome at diagnosis. Using two different classification algorithms, we identified 8 genes to be the most powerful at discriminating between tumors that did or did not experience relapse. Intersecting data from microarrays, high-throughput Fluidigm, and RNA-sequencing, this number of genes was further reduced to *FAM179A* and *FN1.* As FN1 is an ECM key regulator, we suggest that it might be involved in the prognosis and therapeutic response in ALCL, as already suggested in DLBCL.

## Figures and Tables

**Table 1:** Clinical and pathological characteristics of patients and univariate analysis.

		Non_Relapsing Group *n* = 22		Relapsing Group *n* = 26				
						Cox Univariate Analysis		
Characteristics		*n*	%	*n*	%	RR	95% CI	*p*-Value Likehood Ratio
Gender						0.76	0.34–1.69	0.51
	Male	15	68.2	16	61.5			
	Female	7	31.8	10	38.5			
Age (years) ^†^						0.5134	0.23–1.32	0.09302
	Median	15		10				
	Range	6–44		2–50				
St_Jude Stage ^††^						1.405	0.51–3.84	0.49
	I–II	4	18.2	5	19.2			
	III–IV	9	40.9	16	61.5			
Ann Arbor Stage ^††^						1.92	0.80–4.61	0.1266
	I–II	11	50.0	7	26.9			
	III–IV	11	50.0	18	69.2			
IPI score ^††^						2.676	1.002–7.15	0.04183
	0–1	11	50.0	6	23.1			
	2–3	5	22.7	12	46.2			
LDH ^††^						4.46	1.76–11.2	0.00422
	<2 × ULN	20	90.9	15	57.7			
	≥2 × ULN	1	4.5	7	26.9			
Morphological subtype						1.93	0.86–4.35	0.1018
	Common Type	13	59.1	9	34.6			
	SC/LH	9	40.9	17	65.4			
Fusion partner						1.32	0.13–3.2	0.6934
	NPM	20	90.9	24	92.3			
	Others	2	9.1	2	7.7			
Peripheral lymph nodes ^††^								
	No	1	4.5	19	73.1			
	Yes	11	50.0	0	0.0			
Mediastinal involvement ^††^						1.03	0.42–2.55	0.9411
	No	5	22.7	9	34.6			
	Yes	7	31.8	10	38.5			
Visceral involvement (spleen. liver or lung involvement)						2.139	0.98–4.67	0.05414
	No	15	68.2	11	42.3			
	Yes	7	31.8	15	57.7			
Spleen involvement ^††^								
	No	18	81.8	20	76.9			
	Yes	4	18.2	5	19.2			
Liver involvement ^††^								
	No	20	90.9	19	73.1			
	Yes	2	9.1	6	23.1			
Lung involvement ^††^								
	No	18	81.8	14	53.8			
	Yes	4	18.2	11	42.3			
Other Visceral involvement ^††^								
	No	17	77.3	13	50.0			
	Yes	5	22.7	13	50.0			
Skin lesion ^††^						1.11	0.46–2.69	0.8131
	No	18	81.8	17	65.4			
	Yes	4	18.2	7	26.9			
Clinical high risk group ^††^ (spleen or/and liver or/and lung or/and mediastinal involvement or/and skin lesions)						1.13	0.45–2.83	0.7843
	No	4	18.2	6	23.1			
	Yes	12	54.5	20	76.9			
Bone lesions ^††^						0.96	0.33–2.79	0.9344
	No	19	86.4	21	80.8			
	Yes	3	13.6	4	15.4			
Bone marrow involvement ^††^						1.062	0.41–2.83	0.9049
	No	17	77.3	21	80.8			
	Yes	3	13.6	4	15.4			
CNS involvement ^††^						1.172	0.16–8.68	0.8793
	No	21	95.5	24	92.3			
	Yes	1	4.5	1	3.8			
Soft tissue mass ^††^						2.53	0.594–10.78	0.2676
	No	21	95.5	23	88.5			
	Yes	1	4.5	2	7.7			
CD3 positivity ^††^						0.73	0.27–1.97	0.53
	Negative	14	63.6	19	73.1			
	Positive	6	27.3	5	19.2			
MDD ^††^						10.23	1.34–78.02	0.001735
	Negative	6	27.3	1	3.8			
	Positive	3	13.6	17	65.4			

Abbreviations: IPI, international prognostic index; LDH, lactate dehydrogenase; Visceral involvement: lung, liver, spleen; CNS, central nervous system; MDD, minimal disseminated disease; PFS, progression free survival; CI, confidence interval; *p*, *p* value; RR, Relative Risk. ^†^: groups defined by the following criteria: ≥ or < median age (12.5 years). ^††^: Missing Data: St-Jude Stage *n* = 14, Ann Arbor Stage *n* = 1, IPI score *n* = 14, LDH *n* = 5, peripheral lymph nodes *n* = 17, mediastinum *n* = 17, spleen *n* = 1, liver *n* = 1, lungs *n* = 1, other visceral involvement *n* = 1, skin lesions *n* = 2, clinical high risk group *n* = 6, bone lesions *n* = 1, bone marrow involvement *n* = 3, CNS involvement *n* = 1, soft tissue mass *n* = 1, CD3 *n* = 4, MDD *n* = 21.

**Table 2:** Expression levels, fold change (FC), *p*-values, and rank of importance of the genes discriminating relapsing ALK^+^ and non-relapsing ALK^+^ tumors using microarray, high-throughput qPCR, and RNA sequencing data.

		Microarray HG-U133-Plus2.0						Fluidigm Data										RNAseq Kallisto/Sleuth DE				
		Mean Log 2 Intensity						Mean (-Delta)Cq				Wilcoxon		Correlation Microarray-Fluidigm				Sleuth Wald Test			Mean Expression (tpm)	
ProbeSet	GeneSymbol	NR	R	FC R vs. NR	logFC R vs. NR	*p* Value	Adjusted *p* Value (BH)	Mean No_Relapse	Mean Relapse	FC R vs. NR	logFC R vs. NR	*p* Value	Adjusted *p* Value (BH)	Pearson Correlation r	r²	*p* Value	Corresp. ENSG	*p* Value	Adjusted *p* Value (BH)	b (Effect Size ~ logFC Estimator) R vs. NR	Mean No_Relapse	Mean Relapse
228471_at	ANKRD44	7.71	8.76	2.07	1.05	1.08 × 10^−3^	3.11 × 10^−2^	0.4	1.09	1.61	0.69	8.97 × 10^−2^	1.01 × 10^−1^	0.86	0.74	1.98 × 10−^15^	ENSG00000065413.20	1.08 × 10^−2^	1.55 × 10^−1^	0.55		
210031_at	CD247	7.93	9.07	2.21	1.14	2.10 × 10^−3^	4.53 × 10^−2^										ENSG00000198821.11	3.76 × 10^−4^	2.59 × 10^−2^	0.9	30.21	54.51
222043_at	CLU	9.4	10.47	2.09	1.07	1.59 × 10^−3^	3.87 × 10^−2^	5.21	5.9	1.61	0.69	6.67 × 10^−2^	8.12 × 10^−2^	0.88	0.77	1.04 × 10^−16^	ENSG00000120885.22	2.05 × 10^−2^	2.17 × 10^−1^	0.56		
**236717_at**	**FAM179A**	6.31	8.07	3.38	1.76	3.62 × 10^−5^	3.52 × 10^−3^	−1.70	−0.16	2.9	1.54	2.61 × 10^−3^	**1.07 × 10^−2^**	**0.97**	0.93	9.46 × 10^−29^	ENSG00000189350.13	6.80 × 10^−5^	**3.64 × 10^−3^**	**1.23**	24.44	58.53
**205718_at**	**ITGB7**	7.13	8.6	2.77	1.47	1.78 × 10^−3^	4.14 × 10^−2^	−0.36	1.07	2.7	1.43	5.35 × 10^−3^	**1.34 × 10^−2^**	**0.97**	0.95	7.59 × 10^−31^	ENSG00000139626.16	1.55 × 10^−4^	**1.88 × 10^−2^**	**1**	22.92	47.71
1558459_s_at	LOC401320	5.47	6.49	2.03	1.02	1.64 × 10^−9^	1.13 × 10^−5^	−1.51	−1.02	1.4	0.49	6.26 × 10^−2^	7.82 × 10^−2^	0.62	0.38	1.71 × 10^−5^						
218202_x_at	MRPL44	3.45	5.69	4.73	2.24	1.08 × 10^−36^	1.55 × 10^−32^	1.29	1.39	1.07	0.1	1.23 × 10^−1^	1.32 × 10^−1^	0.21	0.05	7.81 × 10^−2^	ENSG00000135900.4	NA	NA	NA		
**213733_at**	**MYO1F**	8.71	9.75	2.06	1.04	2.51 × 10^−5^	2.75 × 10^−3^	0.68	1.65	1.95	0.96	2.43 × 10^−3^	**1.07 × 10^−2^**	**0.91**	0.83	1.15 × 10^−18^	ENSG00000142347.19	6.03 × 10^−5^	**1.14 × 10^−2^**	**0.51**	146.59	229.13
212259_s_at	PBXIP1	6.74	7.74	2	1	2.49 × 10^−3^	4.99 × 10^−2^	0.88	1.72	1.79	0.84	3.67 × 10^−2^	5.51 × 10^−2^	0.95	0.9	2.43 × 10^−25^	ENSG00000163346.17	1.90 × 10^−4^	1.99 × 10^−2^	0.59	15.53	26.77
**206060_s_at**	**PTPN22**	7.49	8.84	2.53	1.34	6.31 × 10^−4^	2.29 × 10^−2^	1.39	2.52	2.18	1.13	4.35 × 10^−3^	**1.22 × 10^−2^**	**0.93**	0.87	3.21 × 10^−22^	ENSG00000134242.16	2.70 × 10^−5^	**1.79 × 10^−3^**	**0.9**	43.82	93.55
208010_s_at	PTPN22	5.99	7.35	2.55	1.35	1.18 × 10^−3^	3.26 × 10^−2^	1.39	2.52	2.18	1.13	4.35 × 10^−3^	**1.22 × 10^−2^**	**0.87**	0.75	8.39 × 10^−16^						
236539_at	PTPN22	7.34	8.45	2.16	1.11	1.49 × 10^−3^	3.74 × 10^−2^	1.39	2.52	2.18	1.13	4.35 × 10^−3^	**1.22 × 10^−2^**	**0.94**	0.89	9.00 × 10^−24^						
218394_at	ROGDI	5.22	6.32	2.13	1.09	3.09 × 10^−9^	1.78 × 10^−5^	−3.38	−2.75	1.55	0.63	6.50 × 10^−3^	**1.54 × 10^−2^**	**0.76**	0.58	2.13 × 10^−10^	ENSG00000067836.13	7.75 × 10^−4^	3.77 × 10^−2^	0.42	6.88	10.24
227552_at	SEPT1	6.36	7.52	2.23	1.15	2.32 × 10^−3^	4.82 × 10^−2^	−0.50	0.68	2.26	1.18	5.05 × 10^−2^	6.89 × 10^−2^	0.93	0.87	3.74 × 10^−22^	ENSG00000180096.12	1.51 × 10^−3^	5.48 × 10^−2^	0.64		
223044_at	SLC40A1	10.78	12.03	2.38	1.25	2.57 × 10^−4^	1.38 × 10^−2^	1.19	2.54	2.55	1.35	1.39 × 10^−3^	7.20 × 10^−3^	0.94	0.89	5.92 × 10^−24^	ENSG00000138449.11	1.21 × 10^−1^	4.94 × 10^−1^	−0.46		
244716_x_at	TMIGD2	6.92	8.89	3.93	1.97	1.42 × 10^−10^	1.28 × 10^−7^	−2.28	−0.76	2.86	1.52	3.30 × 10^−2^	5.13 × 10^−2^	0.74	0.55	1.04 × 10^−9^	ENSG00000167664.8	4.47 × 10^−4^	2.80 × 10^−2^	1.31	19.25	37.27
226997_at	ADAMTS12	6.88	5.79	0.47	−1.08	3.61 × 10^−4^	1.68 × 10^−2^	−0.78	−1.46	0.63	−0.68	4.15 × 10^−2^	5.84 × 10^−2^	0.91	0.82	5.00 × 10^−19^	ENSG00000151388.11	4.53 × 10^−4^	2.80 × 10^−2^	−0.56	5.49	3.14
	ADAMTS12																ENSG00000281690.2	2.84 × 10^−2^	2.54 × 10^−1^	−0.45	7.05	4.54
**224694_at**	**ANTXR1**	8.75	6.95	0.29	−1.80	7.56 × 10^−5^	6.01 × 10^−3^	1.14	−0.17	0.41	−1.30	7.37 × 10^−3^	**1.66 × 10^−2^**	**0.96**	0.91	2.24 × 10^−26^	ENSG00000169604.20	1.15 × 10^−5^	**1.14 × 10^−3^**	**−1.18**	14.29	4.45
204345_at	COL16A1	8.15	6.98	0.45	−1.17	1.16 × 10^−3^	3.24 × 10^−2^	0.78	0.05	0.6	−0.73	5.30 × 10^−2^	7.00 × 10^−2^	0.94	0.88	6.02 × 10^−23^	ENSG00000084636.18	5.14 × 10^−3^	1.08 × 10^−1^	−0.75		
221730_at	COL5A2	10.87	9.71	0.45	−1.16	6.60 × 10^−4^	2.33 × 10^−2^	2.75	1.95	0.57	−0.80	3.85 × 10^−2^	5.59 × 10^−2^	0.92	0.85	1.32 × 10^−20^	ENSG00000204262.14	5.87 × 10^−3^	1.13 × 10^−1^	−0.54		
**225681_at**	**CTHRC1**	11.62	10.05	0.34	−1.57	4.40 × 10^−4^	1.89 × 10^−2^	1.39	0.07	0.4	−1.32	3.89 × 10^−3^	**1.17 × 10^−2^**	**0.97**	0.94	1.69 × 10^−30^	ENSG00000164932.13	8.87 × 10^−8^	**3.25 × 10^−4^**	**−0.78**	29.99	13.22
202450_s_at	CTSK	10.12	8.8	0.4	−1.32	1.37 × 10^−3^	3.59 × 10^−2^	2.25	1.28	0.51	−0.97	1.34 × 10^−2^	2.32 × 10^−2^	0.96	0.92	1.14 × 10^−26^	ENSG00000143387.14	NA	NA	NA		
**201893_x_at**	**DCN**	12.14	10.87	0.42	−1.27	1.37 × 10^−3^	3.59 × 10^−2^	3.98	2.75	0.43	−1.22	3.78 × 10^−3^	**1.17 × 10^−2^**	**0.95**	0.9	2.71 × 10^−25^	ENSG00000011465.18	8.81 × 10^−5^	**1.37 × 10^−2^**	**−1.01**	319.12	132.97
211896_s_at	DCN	12.13	10.72	0.37	−1.42	1.08 × 10^−3^	3.11 × 10^−2^	3.98	2.75	0.43	−1.22	3.78 × 10^−3^	**1.17 × 10^−2^**	**0.94**	0.88	3.10 × 10^−23^						
211813_x_at	DCN	11.66	10.12	0.34	−1.55	4.38 × 10^−4^	1.89 × 10^−2^	3.98	2.75	0.43	−1.22	3.78 × 10^−3^	**1.17 × 10^−2^**	**0.93**	0.86	1.51 × 10^−21^						
201325_s_at	EMP1	8.84	7.83	0.5	−1.01	8.35 × 10^−4^	2.70 × 10^−2^	1.88	1.04	0.56	−0.84	3.78 × 10^−3^	1.17 × 10^−2^	0.91	0.83	9.38 × 10^−20^	ENSG00000134531.10	3.03 × 10^−4^	2.36 × 10^−2^	−0.58	78.54	40.11
201324_at	EMP1	10.77	9.74	0.49	−1.03	8.68 × 10^−5^	6.61 × 10^−3^	1.88	1.04	0.56	−0.84	3.78 × 10^−3^	**1.17 × 10^−2^**	**0.92**	0.85	1.76 × 10^−20^						
**209955_s_at**	**FAP**	8.28	6.44	0.28	−1.84	5.71 × 10^−4^	2.21 × 10^−2^	0.86	−0.81	0.31	−1.67	8.08 × 10^−4^	**5.35 × 10^−3^**	**0.98**	0.95	1.23 × 10^−32^	ENSG00000078098.14	9.08 × 10^−5^	**4.08 × 10^−3^**	**−1.25**	46.2	14.03
**211719_x_at**	**FN1**	12.69	11.33	0.39	−1.36	2.04 × 10^−4^	1.18 × 10^−2^	4.68	3.17	0.35	−1.51	2.12 × 10^−4^	**2.54 × 10^−3^**	**0.98**	0.96	5.68 × 10^−34^	ENSG00000115414.21	1.45 × 10^−5^	**1.27 × 10^−3^**	**−1.18**	680.6	193.53
214701_s_at	FN1	6.29	4.67	0.33	−1.62	5.11 × 10^−7^	1.50 × 10^−4^	4.68	3.17	0.35	−1.51	2.12 × 10^−4^	**2.54 × 10^−3^**	**0.73**	0.53	3.20 × 10^−9^						
210495_x_at	FN1	12.29	10.62	0.31	−1.67	7.05 × 10^−5^	1.07 × 10^−3^	4.68	3.17	0.35	−1.51	2.12 × 10^−4^	**2.54 × 10^−3^**	**0.98**	0.96	3.57 × 10^−34^						
216442_x_at	FN1	12.33	10.65	0.31	−1.69	9.99 × 10^−5^	1.35 × 10^−3^	4.68	3.17	0.35	−1.51	2.12 × 10^−4^	**2.54 × 10^−3^**	**0.98**	0.97	1.58 × 10^−35^						
212464_s_at	FN1	12.32	10.62	0.31	−1.69	1.09 × 10^−5^	1.41 × 10^−3^	4.68	3.17	0.35	−1.51	2.12 × 10^−4^	**2.54 × 10^−3^**	**0.98**	0.96	1.53 × 10^−34^						
**225481_at**	**FRMD6**	8.43	7.29	0.45	−1.14	4.17 × 10^−4^	1.84 × 10^−2^	0.31	−0.58	0.54	−0.89	1.19 × 10^−2^	**2.23 × 10^−2^**	**0.94**	0.88	7.77 × 10^−22^	ENSG00000139926.16	8.55 × 10^−5^	**4.08 × 10^−3^**	**−0.80**	26.78	47.71
225464_at	FRMD6	8.41	7.27	0.45	−1.14	3.71 × 10^−4^	1.69 × 10^−2^	0.31	−0.58	0.54	−0.89	1.19 × 10^−2^	**2.23 × 10^−2^**	**0.93**	0.87	1.20 × 10^−20^						
**227070_at**	**GLT8D2**	8.2	6.95	0.42	−1.25	2.01 × 10^−3^	4.44 × 10^−2^	−0.78	−1.69	0.53	−0.91	2.54 × 10^−2^	**4.09 × 10^−2^**	**0.94**	0.89	7.62 × 10^−22^	ENSG00000120820.12	5.54 × 10^−5^	**3.20 × 10^−3^**	**−0.71**	15.03	7.28
227059_at	GPC6	8.15	6.18	0.25	−1.97	2.46 × 10^−5^	2.75 × 10^−3^	−0.81	−2.77	0.26	−1.96	1.09 × 10^−4^	**2.54 × 10^−3^**	**0.97**	0.94	9.60 × 10^−30^	ENSG00000183098.11	NA	NA	NA		
201035_s_at	HADH	7	5.97	0.49	−1.02	4.44 × 10^−11^	4.56 × 10^−8^	−0.70	−0.86	0.89	−0.16	2.42 × 10^−1^	2.48 × 10^−1^	0.55	0.3	2.93 × 10^−5^	ENSG00000138796.17	2.17 × 10^−2^	2.24 × 10^−1^	−0.21		
226218_at	IL7R	9.7	8.17	0.34	−1.54	3.19 × 10^−4^	1.58 × 10^−2^	1.58	0.49	0.47	−1.09	2.98 × 10^−3^	1.12 × 10^−2^	0.95	0.91	1.27 × 10^−25^	ENSG00000168685.15	4.03 × 10^−4^	2.69 × 10^−2^	−0.59	73.03	39.19
205798_at	IL7R	8.99	7.41	0.33	−1.59	6.31 × 10^−4^	2.29 × 10^−2^	1.58	0.49	0.47	−1.09	2.98 × 10^−3^	**1.12 × 10^−2^**	**0.91**	0.83	1.33 × 10^−19^						
**227140_at**	**INHBA**	9.26	6.5	0.15	−2.76	1.09 × 10^−5^	1.41 × 10^−3^	0.08	−1.94	0.25	−2.01	2.82 × 10^−4^	**2.54 × 10^−3^**	**0.96**	0.91	2.79 × 10^−26^	ENSG00000122641.11	3.05 × 10^−5^	**8.21 × 10^−3^**	**−1.64**	9.2	2.09
**204686_at**	**IRS1**	6.61	5.45	0.45	−1.16	9.09 × 10^−5^	1.27 × 10^−3^	−1.31	−1.77	0.72	−0.47	7.18 × 10^−2^	**8.50 × 10^−2^**	**0.77**	0.59	1.12 × 10^−10^	ENSG00000169047.5	1.37 × 10^−4^	**1.76 × 10^−2^**	**−0.50**	10.16	5.97
204682_at	LTBP2	7.22	6.04	0.44	−1.18	2.44 × 10^−3^	4.92 × 10^−2^	−0.08	−0.98	0.53	−0.91	5.45 × 10^−2^	7.00 × 10^−2^	0.45	0.2	1.03 × 10^−3^	ENSG00000119681.12	5.46 × 10^−5^	1.09 × 10^−2^	−1.02	13.28	5.61
201069_at	MMP2	9.55	7.61	0.26	−1.94	2.12 × 10^−3^	4.57 × 10^−2^	2.23	0.66	0.34	−1.57	1.34 × 10^−2^	2.32 × 10^−2^	0.98	0.96	7.23 × 10^−33^	ENSG00000087245.13	3.56 × 10^−3^	8.81 × 10^−2^	−1.22		
203936_s_at	MMP9	7.78	6.22	0.34	−1.56	2.24 × 10^−3^	4.72 × 10^−2^	0.94	−0.58	0.35	−1.52	5.01 × 10^−3^	1.33 × 10^−2^	0.96	0.91	7.85 × 10^−26^	ENSG00000100985.7	6.81 × 10^−3^	1.22 × 10^−1^	−1.03		
**203939_at**	**NT5E**	7.42	5.96	0.36	−1.46	6.32 × 10^−5^	5.20 × 10^−3^	0.05	−0.96	0.5	−1.01	1.44 × 10^−3^	**7.20 × 10^−3^**	**0.95**	0.9	1.05 × 10^−24^	ENSG00000135318.12	6.37 × 10^−5^	**1.15 × 10^−2^**	**−0.62**	6.52	3.31
204992_s_at	PFN2	8.19	6.8	0.38	−1.39	1.30 × 10^−4^	8.64 × 10^−3^										ENSG00000070087.14	9.83 × 10^−3^	1.47 × 10^−1^	−0.46		
**205479_s_at**	**PLAU**	8.91	7.07	0.28	−1.84	3.01 × 10^−5^	3.13 × 10^−3^	1.82	0.5	0.4	−1.32	2.50 × 10^−4^	**2.54 × 10^−3^**	**0.98**	0.95	6.36 × 10^−32^	ENSG00000122861.16	1.91 × 10^−4^	**1.99 × 10^−2^**	**−0.95**	5.89	2.46
210809_s_at	POSTN	12.88	10.81	0.24	−2.07	1.19 × 10^−4^	8.13 × 10^−3^	1.42	−0.38	0.29	−1.80	3.89 × 10^−3^	1.17 × 10^−2^	0.94	0.88	2.33 × 10^−23^	ENSG00000133110.15	2.52 × 10^−4^	2.17 × 10^−2^	−1.37	292.37	75.76
1555778_a_at	POSTN	11.22	8.86	0.19	−2.36	2.37 × 10^−4^	1.29 × 10^−2^	1.42	−0.38	0.29	−1.80	3.89 × 10^−3^	1.17 × 10^−2^	0.91	0.82	3.07 × 10^−19^						
202975_s_at	RHOBTB3	8.07	7.06	0.5	−1.01	9.35 × 10^−4^	2.88 × 10^−2^	1.33	0.97	0.78	−0.36	3.26 × 10^−1^	3.26 × 10^−1^	0.67	0.44	1.49 × 10^−7^	ENSG00000164292.13	3.70 × 10^−2^	2.88 × 10^−1^	−0.33		
**212110_at**	**SLC39A14**	9.15	7.88	0.41	−1.28	1.43 × 10^−4^	9.34 × 10^−3^	1.48	0.78	0.61	−0.70	1.17 × 10−2	**2.23 × 10−2**	**0.94**	0.89	1.03 × 10^−23^	ENSG00000104635.15	7.67 × 10^−5^	**1.28 × 10^−2^**	**−0.74**	24.89	11.29
**212354_at**	**SULF1**	10.36	8.44	0.26	−1.92	1.82 × 10^−4^	1.10 × 10^−2^	2.23	0.5	0.3	−1.73	2.30 × 10^−4^	**2.54 × 10^−3^**	**0.98**	0.95	1.47 × 10^−32^	ENSG00000137573.14	6.56 × 10^−8^	**3.25 × 10^−4^**	**−1.34**	131.96	37.09
212344_at	SULF1	9.24	7.15	0.24	−2.09	1.06 × 10^−4^	7.35 × 10^−3^	2.23	0.5	0.3	−1.73	2.30 × 10^−4^	**2.54 × 10^−3^**	**0.95**	0.9	4.63 × 10^−25^						
212353_at	SULF1	10.33	8.19	0.23	−2.14	6.30 × 10^−5^	5.20 × 10^−3^	2.23	0.5	0.3	−1.73	2.30 × 10^−4^	**2.54 × 10^−3^**	**0.98**	0.97	2.01 × 10^−36^						
206506_s_at	SUPT3H	6.28	5.26	0.49	−1.02	2.36 × 10^−5^	5.22 × 10^−4^	−1.11	−1.26	0.9	−0.15	1.39 × 10^−1^	1.45 × 10^−1^	0.43	0.18	1.36 × 10^−3^	ENSG00000196284.17	2.45 × 10^−2^	2.37 × 10^−1^	−0.20		
203083_at	THEM4	7.5	6.46	0.39	−1.35	1.86 × 10^−5^	2.19 × 10^−3^	−10.85	−18.80	4.04 × 10−3	−7.95	1.14 × 10^−1^	1.25 × 10^−1^	0.68	0.46	8.14 × 10^−8^	ENSG00000159445.13	3.52 × 10^−3^	8.78 × 10^−2^	−0.32		
**1553118_at**	**THBS2**	9.82	8.47	0.49	−1.04	1.93 × 10^−3^	4.33 × 10^−2^	2.3	1.35	0.52	−0.95	1.83 × 10^−2^	**3.05 × 10^−2^**	**0.98**	0.95	3.75 × 10^−32^	ENSG00000186340.16	3.38 × 10^−5^	**8.78 × 10^−3^**	**−1.00**	70.74	28.41
219410_at	TMEM45A	8.55	7.1	0.37	−1.45	7.23 × 10^−4^	2.49 × 10^−2^	−0.67	−1.71	0.49	−1.04	7.99 × 10^−3^	**1.71 × 10^−2^**	**0.97**	0.93	9.69 × 10^−29^	ENSG00000181458.10	2.32 × 10^−2^	2.30 × 10^−1^	−0.19		
220968_s_at	TSPAN9	5.94	4.73	0.43	−1.21	5.79 × 10^−14^	9.26 × 10^−11^	−0.13	−0.68	0.68	−0.55	9.13 × 10^−3^	**1.87 × 10^−2^**	**0.71**	0.5	1.11 × 10^−8^	ENSG00000011105.14	5.59 × 10^−4^	3.15 × 10^−2^	−0.45	11.05	6.43
243526_at	WDR86	6.21	4.96	0.42	−1.25	4.04 × 10^−7^	1.24 × 10^−4^	−3.20	−3.93	0.61	−0.72	7.69 × 10^−2^	8.87 × 10^−2^	0.64	0.41	5.71 × 10^−7^	ENSG00000187260.16	1.87 × 10^−2^	2.07 × 10^−1^	−0.41		

## Data Availability

The data presented in this study are available on request from the corresponding author.

## Supplementary Materials

The following are available online at https://www.mdpi.com/2072-6694/13/21/5523/s1, Figure S1: Correlation (R²) between gene expression levels in samples, determined by microarray or standard RT-qPCR and compared to high-throughput RT-qPCR, Figure S2: Validation of the 3-genes associated with relapse using Cohort B samples with standard RT-qPCR, Figure S3: Venn diagram showing intersection between the 168 differentially expressed (DE) genes (Sleuth DE_WT) by RNA sequencing, the 47 significantly discriminating genes by the microarray technique and the 29 DE genes validated by high-throughput qPCR, Figure S4: Five genes overexpressed in Relapse group by RNA sequencing, Figure S5: Fifteen genes overexpressed in No-Relapse group by RNA sequencing. Table S1: Overview of clinical ALCL cases used in microarray (all cases; *n* = 48) and RNA sequencing (bold cases; *n* = 39) technologies, Table S2: List of high throughput and standard qPCR primers, Table S3: Functional Enrichment Analysis of the differentially-expressed genes between the relapsing and the no relapsing groups using microarray data, Table S4: Mean gene expression values and fold-changes of the 3-gene classifier measured by high-throughput RT-qPCR, standard RT-qPCR and RNA sequencing in all specimens., Table S5: With a corrected *p*-value < 0.02, 62 genes were found as differentially expressed between the two groups (relapse and no relapse) with the stringent Likelihood Ratio Test (LRT).
