# Supplementary figures and images for: Gene Expression Signature Associated with Clinical Outcome in ALK-Positive Anaplastic Large Cell Lymphoma
Source: Cancers (Basel). 2021 Nov 3;13(21):5523. doi: 10.3390/cancers13215523 (PMC8582782; doi:10.3390/cancers13215523)

## Slide 1
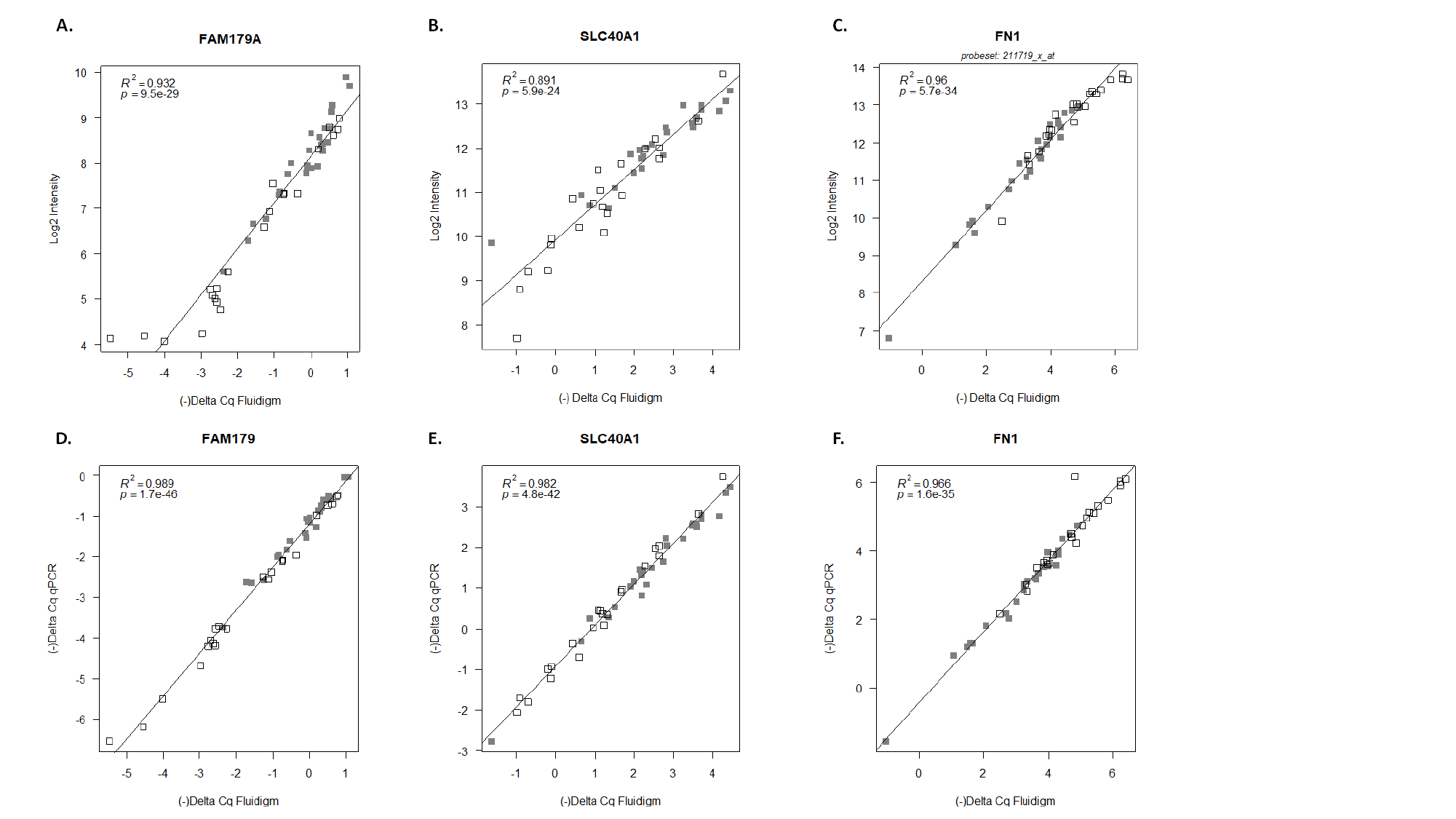

Supplement: Supplementary file 1 [file cancers-13-05523-s001.zip › figure S1.pptx]

## Slide 1
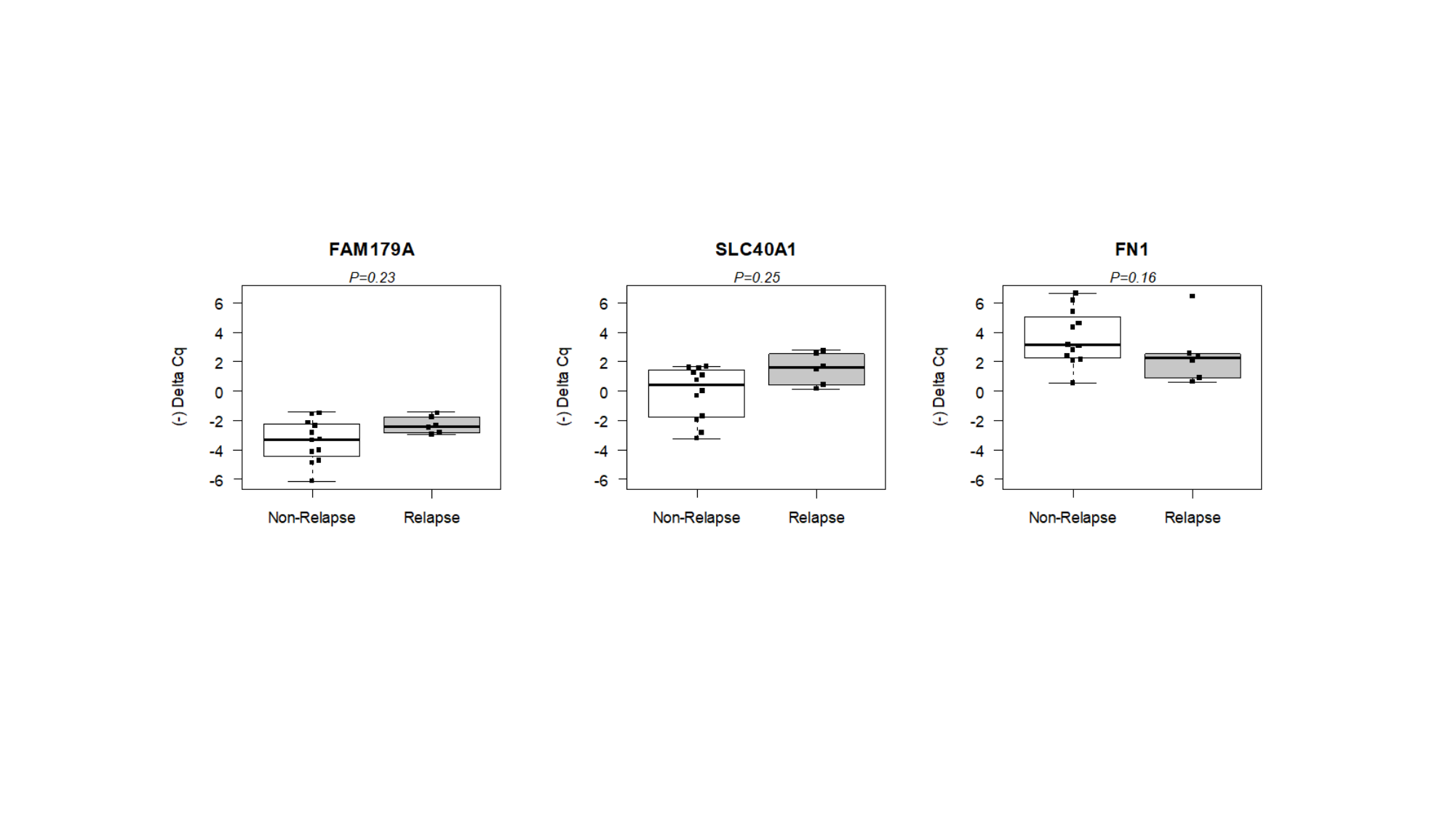

Supplement: Supplementary file 1 [file cancers-13-05523-s001.zip › figure S2.pptx]

## Slide 1
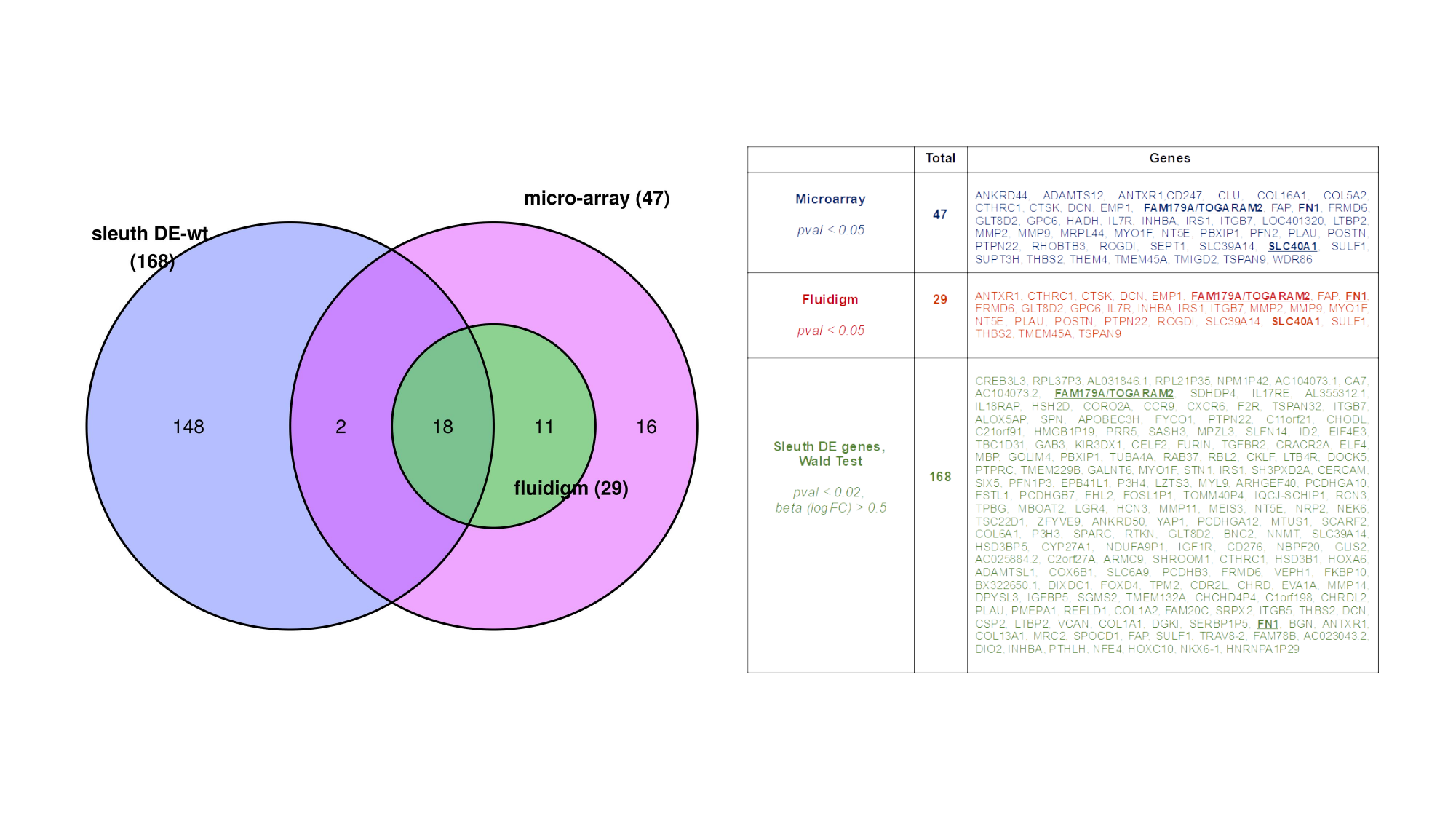

Supplement: Supplementary file 1 [file cancers-13-05523-s001.zip › figure S3.pptx]

## Slide 1
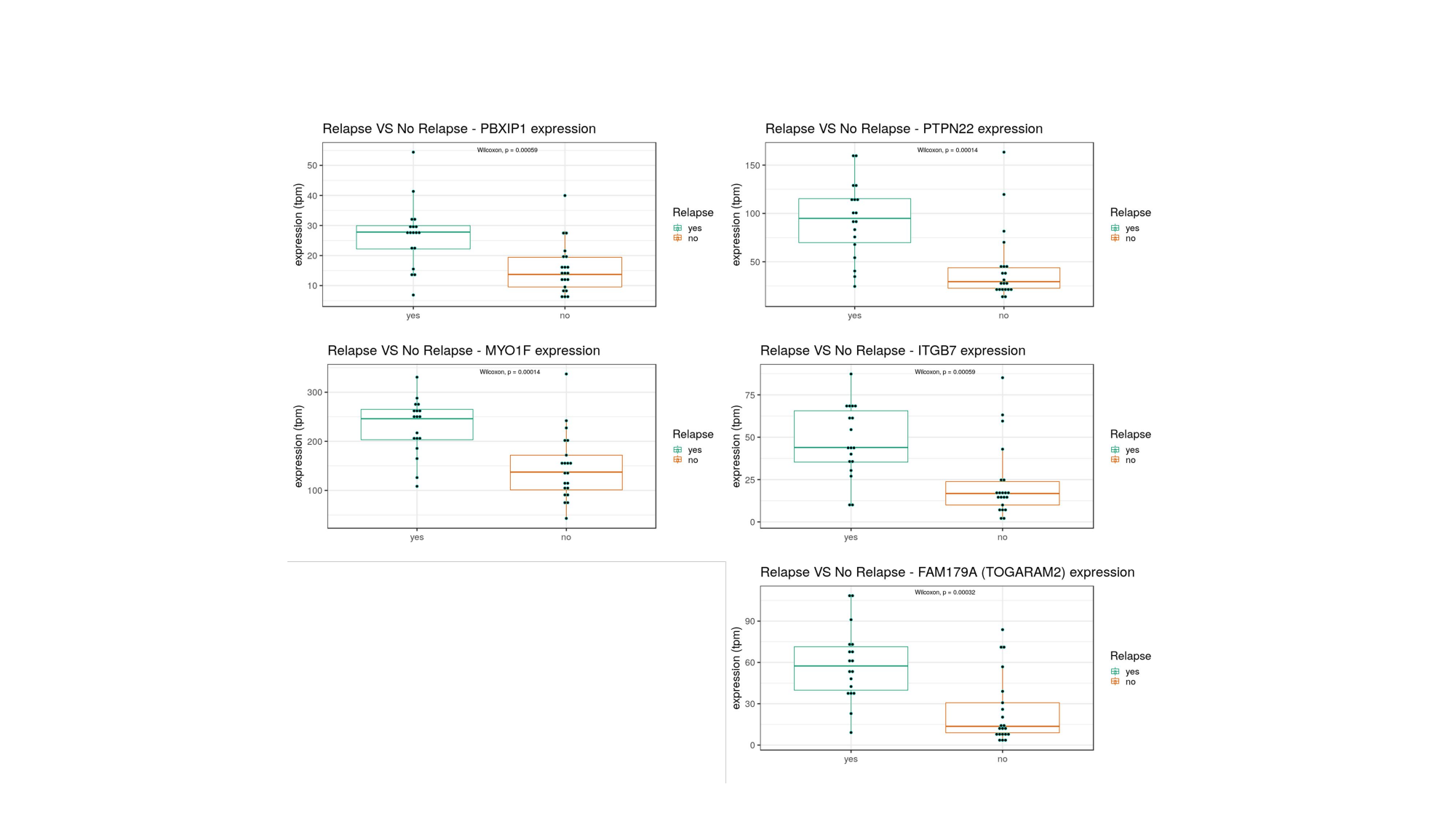

Supplement: Supplementary file 1 [file cancers-13-05523-s001.zip › figure S4.pptx]

## Slide 1
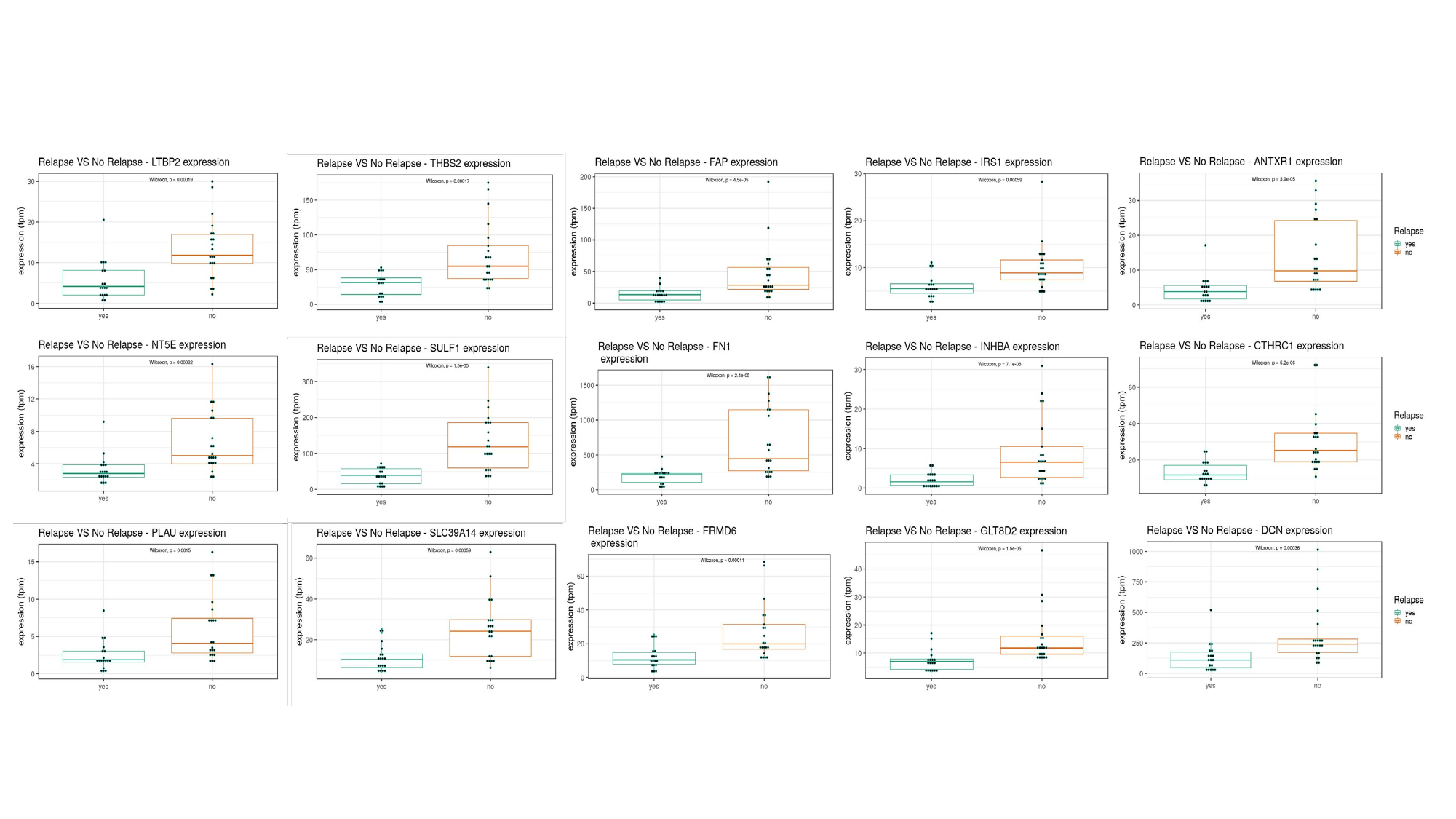

Supplement: Supplementary file 1 [file cancers-13-05523-s001.zip › figure S5.pptx]
